# Increased impairment of cerebral autoregulation in COVID-19 associated pulmonary failure requiring extracorporeal membrane oxygenation

**DOI:** 10.3389/fmed.2024.1423241

**Published:** 2024-07-01

**Authors:** Marcus Thudium, Jochen Kappler, Maximilian J. G. Oremek, Stefan Felix Ehrentraut, Evgeniya Kornilov, Milka Marinova, Christian Putensen, Martin Soehle, Jens-Christian Schewe

**Affiliations:** ^1^Department of Anesthesiology and Intensive Care Medicine, University Hospital Bonn, Bonn, Germany; ^2^Department of Neurobiology, Weizmann Institute of Science, Rehovot, Israel; ^3^Department of Nuclear Medicine, University Hospital Bonn, Bonn, Germany; ^4^Department of Anesthesiology, Intensive Care Medicine and Pain Therapy, University Medical Center Rostock, Rostock, Germany

**Keywords:** ECMO, SARS-CoV-2, ARDS, cerebral autoregulation, NIRS

## Abstract

**Introduction:**

Cerebrovascular complications are feared but also commonly reported in patients with COVID-19 requiring extracorporeal membrane oxygenation (ECMO) support therapy. Besides other reasons, a connection between impaired cerebral autoregulation and SARS-CoV-2 infection as a mechanism for an increase in cerebrovascular complications has been hypothesized.

**Methods:**

In an observational single-center study, we investigated a cohort of 48 patients requiring veno-venous ECMO support therapy with (*n* = 31) and without SARS-CoV-2 infection (*n* = 17). Cerebral autoregulation was assessed with the cerebral oximetry-derived autoregulation index (ORx) based on a moving correlation between arterial pressure and cerebral oximetry.

**Results:**

Patients with ECMO support therapy and SARS-CoV-2 experienced more time with impaired cerebral autoregulation than without SARS-CoV-2 [17 ± 9 vs. 13 ± 9% (*p* = 0.027)]. Patients with SARS-CoV-2 suffering from cerebrovascular complications had more time with impaired autoregulation than non SARS-CoV-2 patients with these complications (19 ± 9 vs. 10 ± 4%, *p* = 0.032).

**Conclusion:**

Our results suggest a connection between SARS-CoV-2 and impaired cerebral autoregulation as well as cerebrovascular complications in SARS-CoV-2 patients.

## 1 Introduction

With the global spreading of the coronavirus pandemic 2019 (COVID-19), a substantial number of patients experienced severe acute respiratory syndrome coronavirus type 2 (SARS-CoV-2) and were admitted to hospitals with acute respiratory distress syndrome (ARDS) (1). For some patients with manifest hypoxemia or hypercarbia requiring mechanical ventilation, veno-venous extracorporeal membrane oxygenation (VV-ECMO) remained a last option in refractory ARDS (2). However, despite its lifesaving properties, cerebral complications such as intracranial hemorrhage or ischemia remain major complications of ECMO therapy. Only recently, it has been reported that the incidence of intracranial hemorrhage was twice as high in SARS-CoV-2 patients undergoing ECMO therapy compared to patients with ARDS due to other reasons (3). Cerebral hyperperfusion and hypercoagulation have also been observed in single cases, the former of which was attributed to impaired cerebral autoregulation due to the SARS-CoV-2 infection (4). Impaired cerebral autoregulation is commonly described as an increased pressure-passive behavior of cerebral blood, thus rendering the brain vulnerable to hypo- and hyperperfusion (5). In patients with severe SARS-CoV-2 infections, both ischemic and hemorrhagic stroke have been reported in the early stages of the pandemic (6). Cerebral autoregulation has been shown to be impaired in a small series of patients with ECMO due to SARS-CoV-2 (7). Brain endothelial cells as well as the blood–brain barrier have been demonstrated to be affected by the virus (8–11). Therefore, an effect of the infection on cerebral autoregulation and possibly on cerebrovascular complications is possible.

In a prospective observational cohort study, we intended to observe cerebral autoregulation properties of patients with ARDS requiring VV-ECMO therapy with and without SARS-CoV-2. We hypothesized an increased impairment of cerebral autoregulation in SARS-CoV-2 patients compared to ARDS due to other causes. Although there is at the moment no uniform representation of intact cerebral autoregulation, a method using a moving Pearson correlation coefficient (ORx) between mean arterial pressure (MAP) and cerebral oximetry with near-infrared spectroscopy (NIRS) has been described previously on several occasions (12). We intentionally chose patients with ECMO therapy since we expected significant percentages of impaired cerebral autoregulation in this critical state.

## 2 Materials and methods

This study was approved by the University of Bonn ethics committee (No. 020/21). Informed consent was waived since both advanced hemodynamic measurements and NIRS represent clinical routine in ARDS patients with ECMO support. Inclusion criteria were: ARDS with VV-ECMO, NIRS monitoring, and calibrated pulse contour analysis. Exclusion criteria were: age under 18 years, pregnancy, veno-arterial ECMO, missing data of NIRS or pulse contour monitoring, < 12 h of measurement time, >50% missing values in NIRS or hemodynamic data during measurement time.

### 2.1 Initiation of ECMO support therapy and monitoring

The setup of our interprofessional ECMO team has been described previously (13). ECMO was either established on our ICU ward or by the ECMO retrieval team at hospitals transferring patients to our ARDS center. Standard cannulation primarily consisted of veno-venous cannulation of the femoral veins. In cases of insufficient blood flow, an additional cannula was inserted into the right jugular vein. Criteria for initiation and the management of ECMO therapy was done in line with the ELSO guidelines (14). After initial stabilization, the ECMO retrieval team transported the patients to the Bonn University Hospital.

For calibrated pulse contour analysis, a 4 or 5F PiCCO catheter (Getinge/Pulsion Medical Systems, Feldkirchen, Germany) was inserted in the femoral or brachial artery of the patient and was attached to a PulsioFlex Monitor (Getinge/Pulsion Medical Systems, Feldkirchen, Germany). Patients received unilateral or bilateral frontal regional cerebral oximetry (rSO_2_) with NIRS (INVOS 5100C/INVOS 7100 cerebral oximeter, Medtronic, Meerbusch, Germany). Patients were included once ECMO as well as PiCCO and NIRS monitoring were established. Data acquisition was temporarily stopped when patients were transferred for CT-Scans and was terminated once ECMO weaning was started. During ECMO therapy, patients were in deep sedation with Isoflurane and Sufentanil and mechanically ventilated using a lung protective ventilation regimen.

Results from routinely performed arterial blood gas analysis were retrieved from the electronic patient file. Maximum and minimum values were recorded for each measurement day.

Cranial computed tomography (cCT) findings were used as a correlate for neurological outcome. CCTs were routinely performed at admission or initiation of ECMO therapy and during ECMO therapy in at least weekly intervals. Triggers for additional brain CT scans were suspected neurological pathologies as revealed by unequal pupil sizes or rSO_2_ decreases. Cerebrovascular complications were represented by cCT pathologies: the presence of intracranial hyperdensities indicative of intracranial hemorrhage, or hypodensities suggestive of cerebral ischemia.

### 2.2 Data analysis

Data were analyzed offline using Matlab R2021b (MathWorks, Natick, United States). A custom-made tool was used for the assessment of cerebral autoregulation: false zero values of NIRS, MAP, and cardiac output were automatically removed and interpolated with linear interpolation. Data were resampled to 5 s intervals. ORx was calculated using a modified version of the Pearson correlation coefficient algorithm. A moving Pearson correlation coefficient was calculated over a time window of 5 min. To remove false low ORx values, the standard deviation of MAP within the ORx window was calculated. ORx values were removed if the change of MAP was less than one standard deviation within the measurement window. In cases of bilateral measurement, the mean of the ORx of both sides was used.

For further analysis of relationships between measurement modalities, the mean and standard deviation of NIRS, MAP, CO, and ORx were computed over 2 min intervals. Negative ORx values were set to zero. ORx values >0.3 were defined to indicate impaired cerebral autoregulation based on previous studies (15). Using this, intervals were grouped into intact and impaired autoregulation. Percentages of total measurement time with impaired autoregulation were calculated for each patient. A linear relationship was tested between NIRS, MAP, and CO with respect to ORx. A Pearson correlation between percentage of ORx above 0.3 and minimum and maximum CO_2_ as well as pH values was calculated.

Statistical comparisons between infection status of patients with respect to overall time with ORx > 0.3 was performed using a Mann–Whitney U Test. Demographic variables of patients (age, gender, APACHE II Score, days of prior hospitalization, length of data recording, and 30-day mortality) were compared using the Mann–Whitney U Test based on disease etiology in an exploratory analysis (SARS-COV-2 vs. non SARS-COV-2).

### 2.3 Sample size estimation

Assuming a cumulative time above the autoregulation threshold (ORx > 0.3) of 40% of total measurement time in the reference group (non-SARS-CoV-2) and of 80% in the test group (SARS CoV-2) with a standard deviation of 40%, a total sample size of 46 is necessary to reach a power of 80% and a two-sided level of significance of 5% under the assumption that the reference group is 0.3 times the size of the test group due to the pandemic. The estimated effect sizes were based on preliminary measurements and previous works on intensive care patients (16, 17). We expected a large drop-out rate due to technical reasons (measurement failures, missing data), therefore we aimed to include 60 patients.

## 3 Results

**Figure 1:**
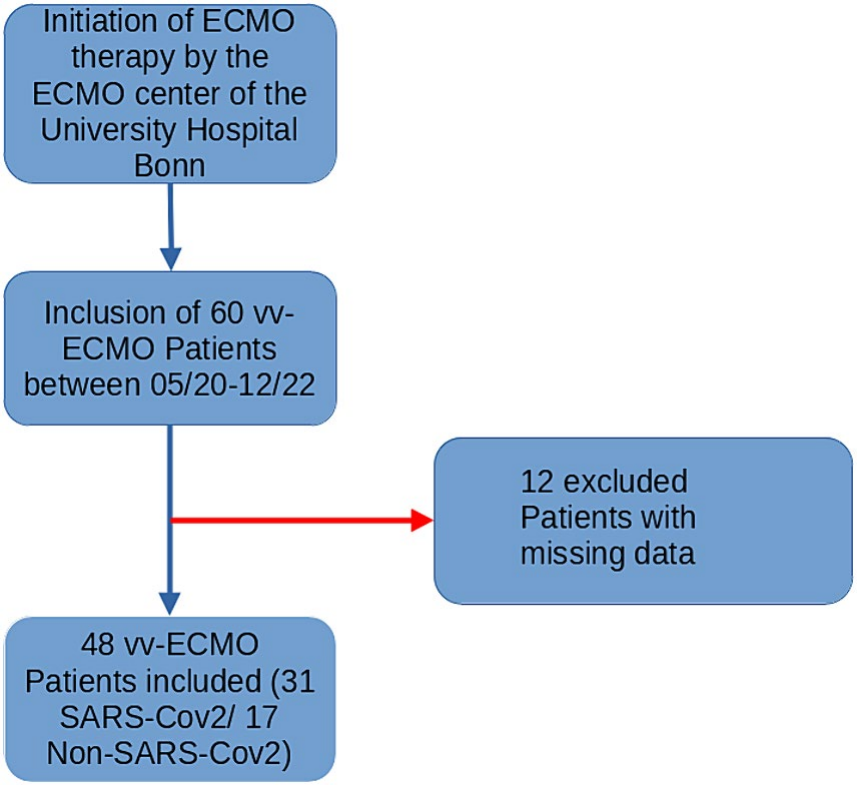
Flowchart of patient inclusion. vv-ECMO, Veno-venous extracorporeal membrane oxygenation.

**Figure 2:**
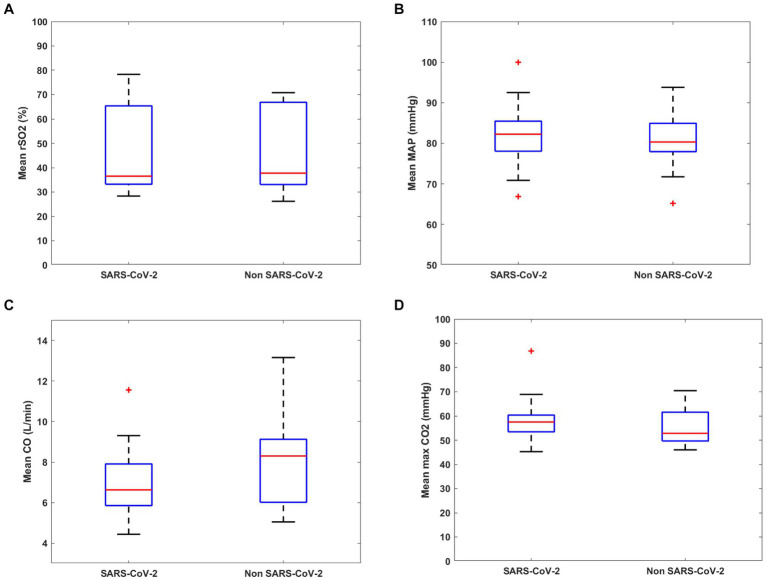
Box-plots of mean hemodynamic, cerebral and ventilation-related parameters in SARS-CoV-2 and non SARS-CoV-2 patients. **(A)** Cerebral oximetry (rSO_2_), **(B)** mean arterial pressure (MAP), **(C)** cardiac output (CO), and **(D)** maximum PaCO_2_. Red crosses represent outliers.

In total, 60 ARDS patients requiring VV-ECMO support were prospectively included during the study period from 05/2020 to 12/2022. 12 patients were excluded due to missing data or recordings shorter than 12 h (Figure 1). Of the remaining 48 patients, 31 had SARS-CoV-2 Infection while 17 had various other causes of ARDS such as bacterial infection, influenza, or secondary ARDS who developed pulmonary failure after sepsis. Demographic data, comorbidities, and disease-related data as well as outcome of included patients are shown in Table 1. Statistical comparison of the included patients grouped by infection etiology showed no significant differences except for APACHE II Score (*p* = 0.024), which indicated a lower disease severity in SARS-CoV-2 patients, while the Charlson Comorbidity Index was equal in both groups. The single items included in the Charlson Comorbidity Index are shown in the Supplementary material. There was also no significant difference in hemodynamic parameters between groups. While overall cerebral saturation was reduced, but there was no difference between groups also shown in Table 1. Figure 2 shows mean hemodynamic values, cerebral oxygenation, and maximal PaCO_2_.

**Table 1:** Demographic and disease-related data.

Parameter	SARS-CoV-2	Non SARS-CoV-2	*p* values
Patients, *n*	31	17	
Age, years	58.3 ± 11.8	51.7 ± 12.7	0.06
APACHE II score	20.8 ± 3.9	23.6 ± 4.0	0.02
CCI, median [IQR]	1.0 [2.0]	1.0 [2.0]	0.97
Female sex, %	25.8	41.2	0.34
Duration of recorded data, h	37.2 ± 28.3	38.9 ± 34.5	0.91
Hospitalization prior to ECMO therapy, days	4.4 ± 3.6	5.5 ± 6.9	0.70
Cerebrovascular complications, %	38.7	35.4	0.83
Cerebral ischemia, *n* (%)	2 (6)	1 (6)	
Intracranial hemorrhage, *n* (%)	10 (32)	2 (12)	
30-day mortality, %	74.2	52.9	0.14
Mean arterial pressure, mmHg, median [IQR]	82.24 [7.41]	80.32 [6.98]	0.58
Cardiac output, L/min, median [IQR]	6.86 [2.05]	8.30[3.10]	0.06
rSO_2_, %, median [IQR]	36.51 [32.15]	37.74 [33.78]	0.89
Maximum arterial CO_2,_ mmHg, median [IQR]	57.5 [6.92]	52.83 [11.88]	0.35

Values are given as mean ± standard deviation. APACHE II, Acute physiology and chronic health evaluation II; CCI, Charlson comorbidity index; ECMO, Extracorporeal membrane oxygenation. Values are reported as mean ± standard deviation where applicable or median [interquartile range, IQR] where indicated.

**Figure 3:**
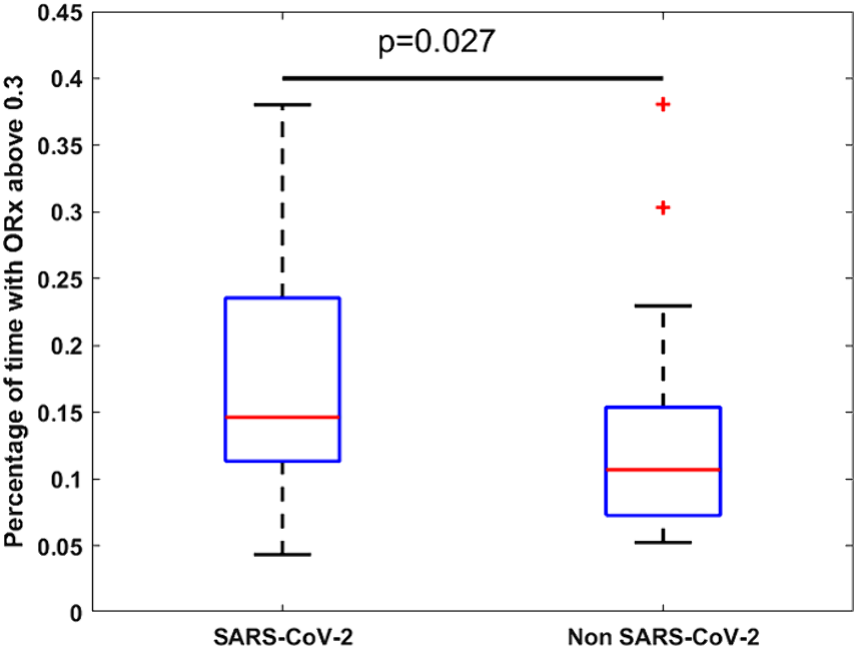
Box-plot of time of ORx indicating impaired cerebral autoregulation in SARS-CoV-2 and non SARS-CoV-2 patients. Time above 0.3 indicates the percentage of total measurement time with an ORx above 0.3 representing impaired autoregulation. Red crosses represent outliers. Orx, Oximetry derived autoregulation index.

**Figure 4:**
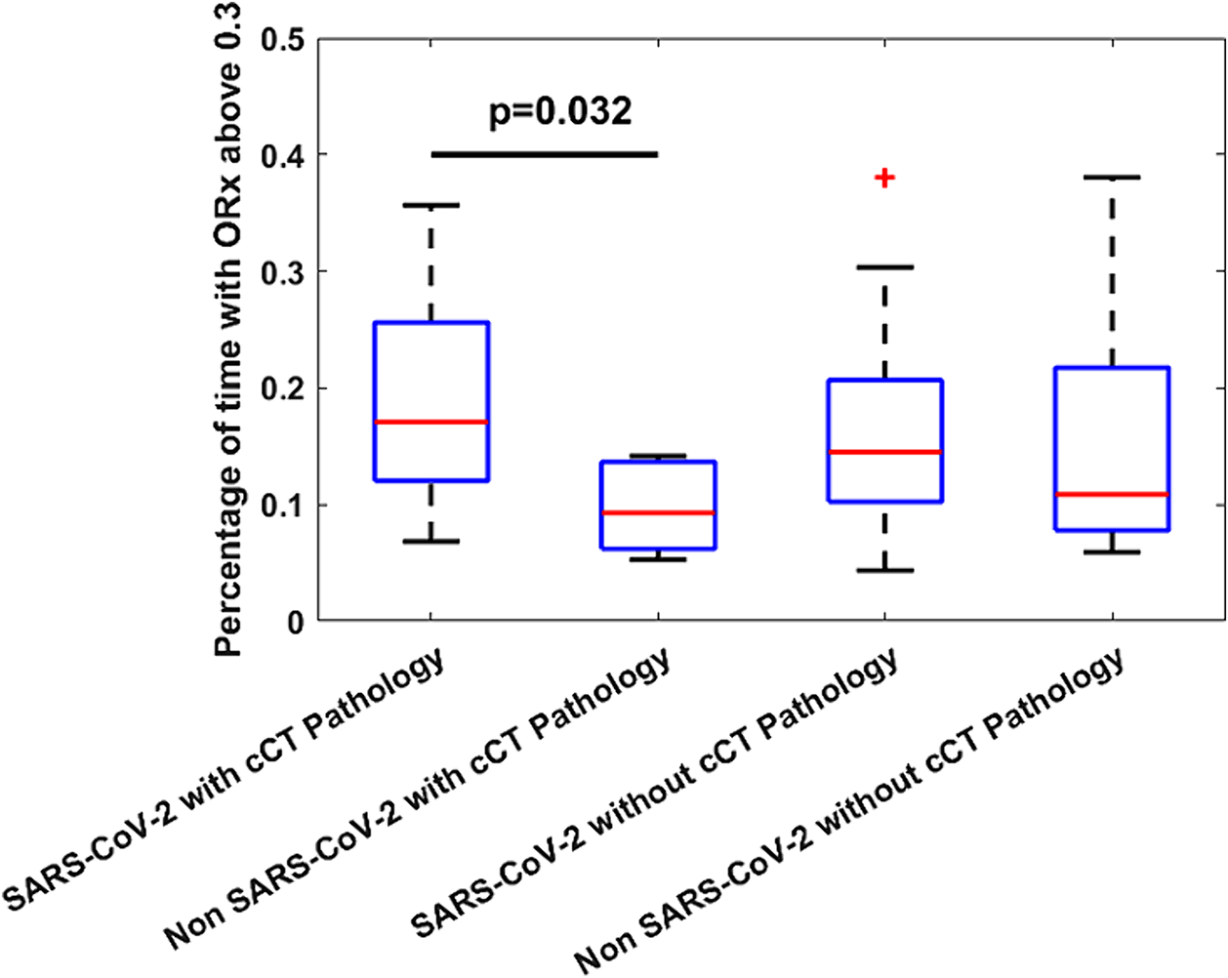
Percentage of ORx indicating dysregulation showing significant difference between SARS-CoV-2 patients with cerebral pathologies and non SARS-CoV-2 patients with cerebral pathologies as found by cCT. Time above 0.3 indicates the percentage of the total measurement time with an ORx above 0.3. The red cross represents an outlier. cCT, Cranial computed tomography; ORx, Oximetry derived autoregulation index.

**Figure 5:**
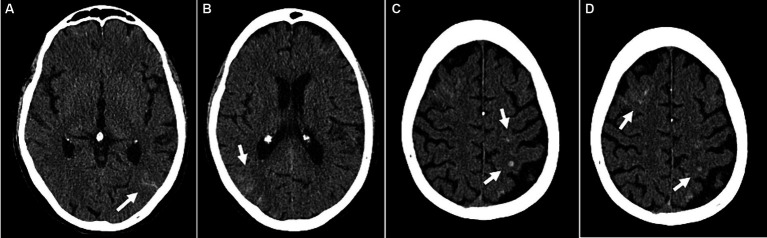
Example of cerebrovascular complications (patient 47): Disseminated small subarachnoid hemorrhages **(A,B)**. Small intracranial hemorrhages in the left parietal and right frontal lobes with adjacent edema **(C,D)**. **(A–D)** Transversal CT images.

Overall, patients experienced impaired cerebral autoregulation as defined by ORx > 0.3 in 15 ± 9% of total recording time. SARS-CoV-2 patients had 17 ± 9% time with impaired autoregulation while in non SARS-CoV-2 patients this was the case in 13 ± 9% (*p* = 0.027) as shown in Figure 3. In patients with SARS-CoV-2 infections vs. patients without, if any cerebrovascular complication was identified by cCT pathologies, an even larger percentage of time with dysregulation was observed (19 ± 9 vs. 10 ± 4%, *p* = 0.032). Cerebral complications in non SARS-CoV-2 patients did not significantly change the amount of time with nonintact autoregulation (10 ± 4 vs. 16 ± 11%, *p* = 0.26; Figure 4; Table 2). Figure 5 shows an example of cCT images with cerebrovascular complications.

**Table 2:** Mann–Whitney U comparison of SARS-CoV-2 vs. non SARS-CoV-2 patients percentage of time with ORx above cerebral autoregulation threshold.

Groups	Mean ± SD (%)	Mean ± SD (%)	*p* value
SARS-CoV-2 vs. nonSARS-CoV-2	17 ± 9	14 ± 9	0.027^*^
SARS-CoV-2 with/without cerebral pathologies	19 ± 9	16 ± 9	0.40^**^
Non SARS-CoV-2 with/without cerebral pathologies	10 ± 4	16 ± 11	0.26^**^
SARS-CoV-2 vs. non SARS-CoV-2 with cerebral pathologies	19 ± 9	10 ± 4	0.032^**^
SARS-CoV-2 vs. non SARS-CoV-2 without cerebral pathologies	16 ± 9	16 ± 11	0.51^**^
SARS-CoV-2 with vs. non SARS-CoV-2 without cerebral pathologies	19 ± 9	15 ± 11	0.23^**^
SARS-CoV-2 without vs. non SARS-CoV-2 with cerebral pathologies	16 ± 9	10 ± 4	0.05^**^

ORx, Oximetry-derived cerebral autoregulation index; SD, Standard deviation. ^*^One-sided significance level < 0.05; ^**^Two-sided significance level < 0.05.

No correlation could be found between ORx and CO, MAP or rSO_2_ in individual patients or based on etiology of the infection. Analysis of arterial blood gases (minimum and maximum CO_2_ and pH over 24 h) revealed no difference between groups, nor was there a relationship between arterial blood gases and ORx. Results of blood gases and APACHE II scores are shown in Supplementary material.

## 4 Discussion

In this prospective observational cohort study, we showed that Patients undergoing VV-ECMO support therapy for SARS-CoV-2 had impaired cerebral autoregulation compared to non SARS-CoV-2 patients. Furthermore, we observed that SARS-CoV-2 patients with cerebrovascular complications had a larger percentage of impaired cerebral autoregulation than non SARS-CoV-2 patients with cerebrovascular pathologies. Our results are in line with previous reports proposing an effect of SARS-CoV-2 infection on the cerebral vasculature (8). A relative increase of ischemic and hemorrhagic stroke in SARS-CoV-2 has been reported previously (3, 6). In this study, we observed an equal incidence of cerebral pathologies in both groups. Impaired cerebral autoregulation has been proposed previously in SARS-CoV-2 infection in single cases and small series (4, 7). Schramm et al. (16) showed that severe sepsis is associated with impaired cerebral autoregulation using a transcranial doppler sonography-derived autoregulation index. Similar results are reported by Crippa et al. (17), showing that 50% of patients with sepsis exhibit impaired cerebral autoregulation which is associated with brain dysfunction. Burzynska et al. (7) reported in a series of 16 SARS-CoV-2 patients with ARDS undergoing vv-ECMO therapy that these patients are likely to experience both elevated biomarkers of cerebral damage and impairment of cerebral autoregulation. We can essentially confirm these findings with the additional finding that there is a difference in cerebral autoregulation between SARS-CoV-2 and non SARS-CoV-2 patients. While SARS-CoV-2 patients had a smaller burden of disease according to APACHE II scores, they still experienced more time with impaired cerebral autoregulation. This again suggests a possible effect of SARS-CoV-2 infection on the cerebral vasculature irrespective of disease severity. The mechanism of autoregulation impairment may be found in the infection of cerebral endothelial cells by the SARS-CoV-2 virus (8). It has been shown that brain endothelial cells are subjected to direct SARS-CoV-2 infection by a flow-mediated expression of angiotensin-converting enzyme 2 (ACE2). SARS-CoV-2 S-Protein binding to ACE2 has been reported to trigger a complex cascade causing the release of inflammatory cytokines and a shift to a prothrombotic state as well as a suppression of NO release and subsequent ACE2 downregulation (8, 18). The following endothelial dysregulation was reported to result in a loss of the endothelium-dependent vasodilator response in severe SARS-CoV-2 infection with increased plasma levels of cytokines and chemokines involved in vascular function and inflammation (19). This has been hypothesized to be the cause for hypoperfusion and stroke in SARS-CoV-2 patients, which was suspected to be associated with impaired cerebral autoregulation (18, 20).

Our results suggest that NIRS-derived autoregulation indices may identify patients at risk of intracranial hemorrhage or ischemic stroke during ECMO therapy. However, to confirm this a larger trial with cCT triggered by ORx values would be required. In non SARS-CoV-2 patients, there is no clear difference between the groups. This may be due to the heterogeneous patient pool used in this study. A larger difference would be expected between the groups in a more homogenous control group.

Apart from the disease, therapy measures may also influence cerebral autoregulation. A relationship of ORx with hemodynamic parameters such as MAP has been reported, but was not seen in our cohort nor was this the case with cardiac output (21). Vasopressor and inotropic therapy at some point of the observation might explain this as well as the overall small percentages of ORx > 0.3 (22). Fanelli et al. (23) reported the restoration of autoregulation after initiation of ECMO therapy. Kahl et al. presented an association between hypocapnia and impaired cerebral autoregulation in SARS-CoV-2 patients while hypercapnia did not have an effect on autoregulation indices. Taccone et al. (24) showed impairment of autoregulation in septic shock, especially in hypercapnia. In our patients, we did not observe an effect of PaCO_2_. Thus, it appears that hemodynamic parameters did not influence ORx significantly, nor was ORx influenced by secondary effects of the disease or the therapy such as CO_2_; rather the impairment of ORx may be seen as an effect of the disease itself.

There are some limitations in this study. First, this is an observational study and results should be interpreted in this context. While an association between cerebrovascular pathologies and ORx could be shown in SARS-CoV-2 patients, it remains difficult to distinguish between cause and effect, therefore a causal link cannot be established in this context. Since the same effect cannot be seen in non SARS-CoV-2 ARDS, an influence of the SARS-CoV-2 appears likely, but cannot be finally confirmed in this setting. A much larger study with homogenous patient collectives would be required. In addition, the time course of ORx in relation to the occurrence of cerebrovascular complications remains unknown. Age and gender may represent confounders, which may possibly affect cerebral autoregulation properties, although this is unlikely in our cohort since no significant difference could be seen between the groups.

In summary, in this study in a cohort of VV-ECMO patients, we could show that SARS-CoV-2 infection is associated with an increased time with ORx above 0.3 indicating impaired cerebral autoregulation. Furthermore, in patients with cerebral pathologies, this effect is more pronounced. No effect of hemodynamic parameters or of PaCO_2_ on ORx could be observed. Further research is needed to investigate the utility of ORx measurements to identify patients at risk of cerebrovascular complications.

In summary, our results suggest that ARDS caused by SARS-CoV-2 is associated with more impaired cerebral autoregulation when compared to non SARS-CoV-2 ARDS patients. Similarly, SARS-CoV-2 patients with cerebrovascular complications have more impaired cerebral autoregulation than non SARS-CoV-2 patients with cerebrovascular complications. Further investigations may allow the early identification of patients at risk of cerebrovascular complications by ORx or the monitoring of such patients.

## Data availability statement

The datasets presented in this study can be found in online repositories. The names of the repository/repositories and accession number(s) can be found below: sciebo, https://uni-bonn.sciebo.de/s/gAfoVOrfxcncdAL.

## Ethics statement

The studies involving humans were approved by University of Bonn ethics committee (No. 020/21). The studies were conducted in accordance with the local legislation and institutional requirements. The ethics committee/institutional review board waived the requirement of written informed consent for participation from the participants or the participants’ legal guardians/next of kin because the study involved routine monitoring only and was focused on data analysis.

## Author contributions

MT: Conceptualization, Investigation, Project administration, Supervision, Validation, Writing – original draft, Writing – review & editing. JK: Data curation, Investigation, Validation, Writing – original draft, Writing – review & editing. MO: Formal analysis, Validation, Visualization, Writing – original draft, Writing – review & editing. SE: Methodology, Writing – original draft, Writing – review & editing. EK: Conceptualization, Methodology, Validation, Writing – original draft, Writing – review & editing. MM: Data curation, Visualization, Writing – original draft, Writing – review & editing. CP: Writing – original draft, Writing – review & editing. MS: Formal analysis, Software, Validation, Writing – original draft, Writing – review & editing. J-CS: Conceptualization, Investigation, Supervision, Validation, Writing – original draft, Writing – review & editing.
