# Supplementary material for: Increased impairment of cerebral autoregulation in COVID-19 associated pulmonary failure requiring extracorporeal membrane oxygenation
Source: Front Med (Lausanne). 2024 Jul 1;11:1423241. doi: 10.3389/fmed.2024.1423241 (PMC11246866; doi:10.3389/fmed.2024.1423241)
Supplement: Supplementary file 2 [file Table_2.DOCX]

| **label** | **variable** | **CoV2** | | **Total** |
| --- | --- | --- | --- | --- |
|  |  | **negative** | **positive** |  |
| myocardial infarction | No | 17 (100%) | 30 (97%) | 47 (98%) |
|  | Yes | 0 (0%) | 1 (3%) | 1 (2%) |
|  | Total | 17 (35%) | 31 (65%) | 48 (100%) |
| congestive heart failure | No | 15 (88%) | 30 (97%) | 45 (94%) |
|  | Yes | 2 (12%) | 1 (3%) | 3 (6%) |
|  | Total | 17 (35%) | 31 (65%) | 48 (100%) |
| peripheral vascular disease | No | 16 (94%) | 30 (97%) | 46 (96%) |
|  | Yes | 1 (6%) | 1 (3%) | 2 (4%) |
|  | Total | 17 (35%) | 31 (65%) | 48 (100%) |
| cerebrovascular disease | No | 17 (100%) | 29 (94%) | 46 (96%) |
|  | Yes | 0 (0%) | 2 (6%) | 2 (4%) |
|  | Total | 17 (35%) | 31 (65%) | 48 (100%) |
| Dementia | No | 17 (100%) | 31 (100%) | 48 (100%) |
|  | Total | 17 (35%) | 31 (65%) | 48 (100%) |
| chronic pulmonary disease | No | 13 (76%) | 21 (68%) | 34 (71%) |
|  | Yes | 4 (24%) | 10 (32%) | 14 (29%) |
|  | Total | 17 (35%) | 31 (65%) | 48 (100%) |
| connective tissue disease | No | 16 (94%) | 29 (94%) | 45 (94%) |
|  | Yes | 1 (6%) | 2 (6%) | 3 (6%) |
|  | Total | 17 (35%) | 31 (65%) | 48 (100%) |
| peptic ulcer diseases | No | 17 (100%) | 31 (100%) | 48 (100%) |
|  | Total | 17 (35%) | 31 (65%) | 48 (100%) |
| mild liver disease | No | 17 (100%) | 31 (100%) | 48 (100%) |
|  | Total | 17 (35%) | 31 (65%) | 48 (100%) |
| moderate to severe liver disease | No | 17 (100%) | 31 (100%) | 48 (100%) |
|  | Total | 17 (35%) | 31 (65%) | 48 (100%) |
| Diabetes | No | 13 (76%) | 23 (74%) | 36 (75%) |
|  | Yes | 4 (24%) | 8 (26%) | 12 (25%) |
|  | Total | 17 (35%) | 31 (65%) | 48 (100%) |
| Diabetes with organ damage | No | 17 (100%) | 31 (100%) | 48 (100%) |
|  | Total | 17 (35%) | 31 (65%) | 48 (100%) |
| Hemiplegia | Yes | 17 (100%) | 31 (100%) | 48 (100%) |
|  | Total | 17 (35%) | 31 (65%) | 48 (100%) |
| severe renal disease | No | 16 (94%) | 27 (87%) | 43 (90%) |
|  | Yes | 1 (6%) | 4 (13%) | 5 (10%) |
|  | Total | 17 (35%) | 31 (65%) | 48 (100%) |
| Tumor | No | 16 (94%) | 28 (90%) | 44 (92%) |
|  | Yes | 1 (6%) | 3 (10%) | 4 (8%) |
|  | Total | 17 (35%) | 31 (65%) | 48 (100%) |
| Leukemia | No | 16 (94%) | 30 (97%) | 46 (96%) |
|  | Yes | 1 (6%) | 1 (3%) | 2 (4%) |
|  | Total | 17 (35%) | 31 (65%) | 48 (100%) |
| Lymphoma | No | 17 (100%) | 30 (97%) | 47 (98%) |
|  | Yes | 0 (0%) | 1 (3%) | 1 (2%) |
|  | Total | 17 (35%) | 31 (65%) | 48 (100%) |
| metastatic solid tumor | No | 16 (94%) | 31 (100%) | 47 (98%) |
|  | Yes | 1 (6%) | 0 (0%) | 1 (2%) |
|  | Total | 17 (35%) | 31 (65%) | 48 (100%) |
| AIDS | No | 16 (94%) | 31 (100%) | 47 (98%) |
|  | Yes | 1 (6%) | 0 (0%) | 1 (2%) |
|  | Total | 17 (35%) | 31 (65%) | 48 (100%) |
